# Lobular panniculitis of the thigh as the only cutaneous manifestation of reactivation of Chagas disease in a renal transplant patient: a case report

**DOI:** 10.1590/0037-8682-0269-2020

**Published:** 2021-03-22

**Authors:** Walmar Roncalli Pereira de Oliveira, Karina Romero-Sandoval, Tiara Souza Magalhães, Marcelo Abrantes Giannotti, Stephen Keith Tyring, Pedro Victor Alcantara da Costa

**Affiliations:** 1 Universidade de São Paulo, Faculdade de Medicina, Hospital das Clínicas, Departamento de Dermatologia, São Paulo, SP, Brasil.; 2 University of Texas Health Science Center, Department of Dermatology. Houston, Texas, United States of America.

**Keywords:** Trypanosomiasis, Reactivation, Transplant, SOTRs

## Abstract

Reactivation of chronic *Trypanosoma cruzi* infection in solid organ transplant recipients (SOTRs) has been reported. The patient presented with a 2-week history of two painful erythematous, infiltrated plaques with central ulceration and necrotic crust on the left thigh. She had a history of chronic indeterminate Chagas disease (CD) and had received a kidney transplant before 2 months. Skin biopsies revealed lobular panniculitis with intracellular amastigote forms of *T. cruzi*. The patient was diagnosed with CD reactivation. Treatment with benznidazole significantly improved her condition. CD reactivation should be suspected in SOTRs living in endemic areas with clinical polymorphism of skin lesions.

## INTRODUCTION

Chagas disease (CD) or American trypanosomiasis is endemic to 21 Latin American countries with 6-7 million affected people. *Trypanosoma cruzi*, the etiological agent of the disease, is transmitted via different routes-through the hematophagous triatomid insect vector, blood transfusions, congenital infections, oral transmission, and organ transplantation^1^ ^,^ ^2^.

In the last few decades, reactivation of chronic *T. cruzi* infection has been observed in immunosuppressed patients, such as those with acquired immunodeficiency syndrome, those undergoing solid organ transplantation, and those undergoing chemotherapeutic treatments. Reactivation of CD is defined as the passage from a chronic or latent state in which the patient is stable to an acute condition with increased levels of parasitemia and serological markers. These patients may develop severe and even lethal forms of the disease, which mandates early diagnosis and prompt treatment^1^ ^,^ ^3^ ^,^ ^4^.

In solid organ transplant recipients (SOTRs), CD reactivation can manifest in different clinical forms such as skin lesions, fever, asthenia, hepatosplenomegaly, cardiomyopathy, and central nervous system involvement. Panniculitis is characterized by the inflammation of subcutaneous fat, which can be confirmed via histopathology that demonstrates the intracellular amastigote form of *T. cruzi* ^1^ ^,^ ^5^ ^,^ ^6^.

We have presented a case of a renal transplant patient who developed lobular panniculitis as the only manifestation of reactivated chronic *T. cruzi* infection. The patient was treated with benznidazole for 30 days; the treatment was well tolerated and led to complete clearance of the lesions. 

## CASE REPORT

**FIGURE 1:**
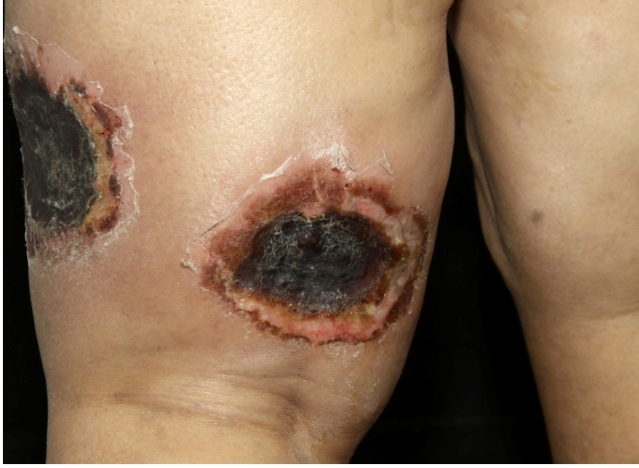
Two erythematous, infiltrated plaques with central ulceration and necrotic crusting approximately 8-10 cm in diameter on the lateral and posterior aspects of the left thigh.

A 51-year-old Caucasian woman was admitted to the Dermatologic Clinic of University of São Paulo, Brazil, with a 2-week history of painful skin lesions. She presented with two erythematous infiltrated plaques with central ulceration and necrotic crusts approximately 8-10 cm in diameter on the lateral and posterior aspects of the left thigh (Figure 1). Asthenia was the only systemic symptom.

**FIGURE 2:**
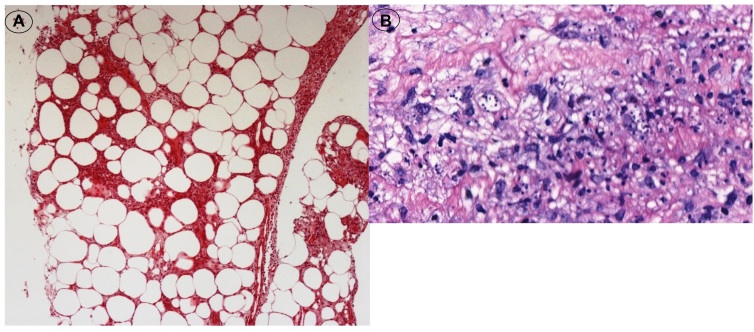
**(A)** Lobular panniculitis characterized by neutrophilic infiltrate permeating adipose tissue; hematoxylin and eosin (HE) stain, 100×. **(B)** Intracellular microorganisms corresponding morphologically to the amastigote form of *Trypanosoma cruzi;* HE stain, 400×.

**FIGURE 3:**
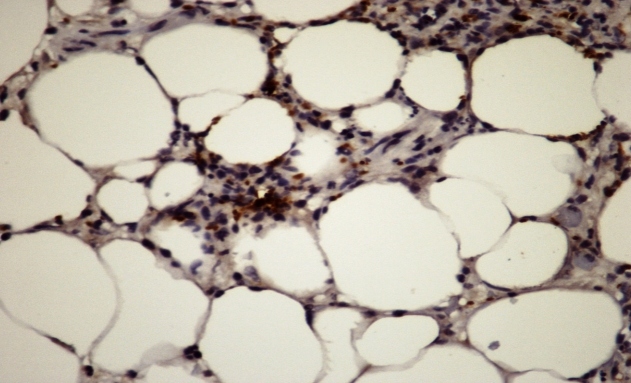
Immunohistochemistry reaction revealing amastigotes (marked by brown chromogen) between inflammatory cells; HE stain, 400×.

The patient had a history of hypertension and chronic indeterminate CD. Two months prior to presentation, she had received a kidney transplant from a deceased donor due to hypertensive nephrosclerosis and was receiving the following immunosuppressive drugs: tacrolimus (6 mg), everolimus (8 mg), prednisone (10 mg), and mycophenolate mofetil (1 g). Blood and urine culture results were negative and chest radiographs and electrocardiograms revealed normal findings. A skin biopsy revealed lobular panniculitis with focal neutrophilic vasculitis and intracellular microorganisms corresponding morphologically to the amastigote form of *T. cruzi* (Figure 2). Immunohistochemical analysis confirmed the presence of *T. cruzi* (Figure 3)*.* Parasites were not found in three fresh blood smears. A qualitative polymerase chain reaction (PCR) assay to detect *T. cruzi* in blood samples showed positive results during post-transplant monitoring. 

**FIGURE 4:**
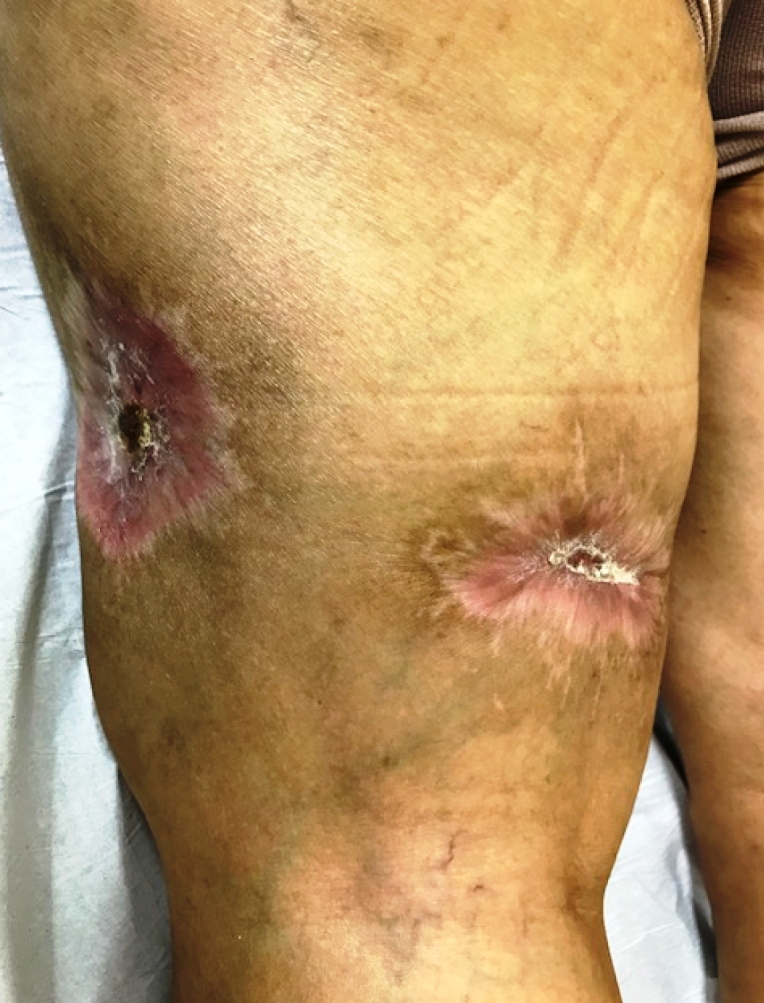
Three months after treatment initiation.

She was treated with benznidazole (5 mg/kg/day) for 30 days, with total clearances of lesions and pain cessation at the 3-month follow-up (Figure 4). Immunosuppressive therapy was not modified. According to the transplantation team, alterations in the immunosuppressive regimen at that point could provoke allograft rejection.

## DISCUSSION

CD reactivation can manifest in a diversity of cutaneous lesions such as erythematous papules, nodules, plaques with necrosis, panniculitis, and/or skin ulcers^1^ ^,^ ^3^ ^-^ ^7^. In SOTRs, skin lesions start to develop from 40 days to 2 years after transplantation, although the incidence is higher during the first year due to intense immunosuppressive therapy^4^. Our patient developed panniculitis as the only manifestation of CD reactivation, which began to appear 45 days after the renal transplantation.

Diagnosis is made by detecting parasitemia or through a biopsy of the compromised organ where the amastigotes can be observed^7^ ^,^ ^8^. Hemocultures and xenodiagnoses are also used, but they have low sensitivity. PCR analyses are more sensitive and specific for diagnosing CD reactivation in the blood and tissue biopsy samples^9^. In our patient, diagnosis was confirmed via histopathological analysis, immunohistochemistry, and qualitative PCR analysis for *T. cruzi* in blood samples.

Although the cardiovascular and central nervous systems are most frequently affected by CD reactivation, the skin was the only organ affected in our patient. Although panniculitis as the only clinical manifestation of CD reactivation is not common, it should be suspected in SOTRs with clinical polymorphism of skin lesions who live in an endemic area, especially those at high risk (previous positive serology and/or chagasic donor)^10^.

There is a lack of evidence regarding post-transplant prophylaxis decreasing the risk of reactivation, and prophylaxis is not routinely administered in transplant centers in Latin America. Nevertheless, some studies recommend careful post-transplantation monitoring using PCR analyses of the peripheral blood for early diagnosis and prompt treatment^11^. Prophylaxis was not performed in our patient, but post-transplant monitoring of the peripheral blood was performed using PCR analysis, which helped to establish the diagnosis. 

Although CD reactivation can be very severe in SOTRs, which might lead to modifications in immunosuppressive therapy such as lowering the doses, reducing the number of immunosuppressants, and/or replacing mycophenolate mofetil by azathioprine^1^ ^,^ ^4^, our patient responded well to treatment without demonstrating the need to make any adjustment in the immunosuppressive regimen. 

Treatment with nifurtimox or benznidazole can be administered for 30-60 days. Benznidazole is suggested as the first choice due to its lower toxicity; however, it can present significant toxicity such as bone marrow suppression and peripheral neuritis^12^. Our patient showed good tolerability to benznidazole (5 mg/kg/day for 30 days), with no side effects and total remission of lesions.
